# Machine Learning Techniques for Personalised Medicine Approaches in Immune-Mediated Chronic Inflammatory Diseases: Applications and Challenges

**DOI:** 10.3389/fphar.2021.720694

**Published:** 2021-09-30

**Authors:** Junjie Peng, Elizabeth C. Jury, Pierre Dönnes, Coziana Ciurtin

**Affiliations:** ^1^ Department of Medicine, Centre for Adolescent Rheumatology Versus Arthritis, University College London, London, United Kingdom; ^2^ Department of Medicine, Centre for Rheumatology Research, University College London, London, United Kingdom; ^3^ Scicross AB, Skövde, Sweden

**Keywords:** machine learning, autoimmune disease, personalised medicine, biomarker, omics

## Abstract

In the past decade, the emergence of machine learning (ML) applications has led to significant advances towards implementation of personalised medicine approaches for improved health care, due to the exceptional performance of ML models when utilising complex big data. The immune-mediated chronic inflammatory diseases are a group of complex disorders associated with dysregulated immune responses resulting in inflammation affecting various organs and systems. The heterogeneous nature of these diseases poses great challenges for tailored disease management and addressing unmet patient needs. Applying novel ML techniques to the clinical study of chronic inflammatory diseases shows promising results and great potential for precision medicine applications in clinical research and practice. In this review, we highlight the clinical applications of various ML techniques for prediction, diagnosis and prognosis of autoimmune rheumatic diseases, inflammatory bowel disease, autoimmune chronic kidney disease, and multiple sclerosis, as well as ML applications for patient stratification and treatment selection. We highlight the use of ML in drug development, including target identification, validation and drug repurposing, as well as challenges related to data interpretation and validation, and ethical concerns related to the use of artificial intelligence in clinical research.

## Introduction

Machine learning (ML) is one subset of artificial intelligence (AI) that aims to build analytical models by learning from existing data. The concept of AI and ML can be traced back to the mid-20th century when building a “machine that can learn from experience” was proposed by mathematician Alan Turing (Turing, 1995). After decades of incremental development and technological innovation, ML has emerged as a powerful discipline for a wide range of scientific research and industrial applications, with a particular strength in discovering patterns in complex, high dimensional data and examining non-linear relationships. In recent years, substantial clinical breakthroughs using ML applications have been made including disease prevention, diagnosis, prognosis, drug discovery and clinical trial design (Stafford et al., 2020; MacEachern and Forkert, 2021). Indeed, the rapid expansion in the availability of patient data has now placed ML under the spotlight for developing data-oriented precision medicine approaches. Immune-mediated inflammatory diseases, such as autoimmune rheumatic diseases (ARDs), inflammatory bowel disease (IBD), immune mediated chronic kidney disease (CKD) and multiple sclerosis (MS), comprise a large group of complex, multifactorial conditions associated with chronic inflammation triggered by dysregulated immune responses. These diseases are highly heterogeneous in presentation, commonly involving multi-organs and systems, and therefore are characterised by complex pathogenic mechanisms and highly variable response to therapies. Thus, applying advanced ML techniques to the clinical study of immune-mediated inflammatory diseases could help develop personalised medicine approaches and improved disease management. In this review, ML applications in clinical research are highlighted and the key challenges and limitations of applying ML towards the goal of personalised medicine in various immune-mediated chronic inflammatory diseases are discussed.

### Types of Machine Learning

**FIGURE 1:**
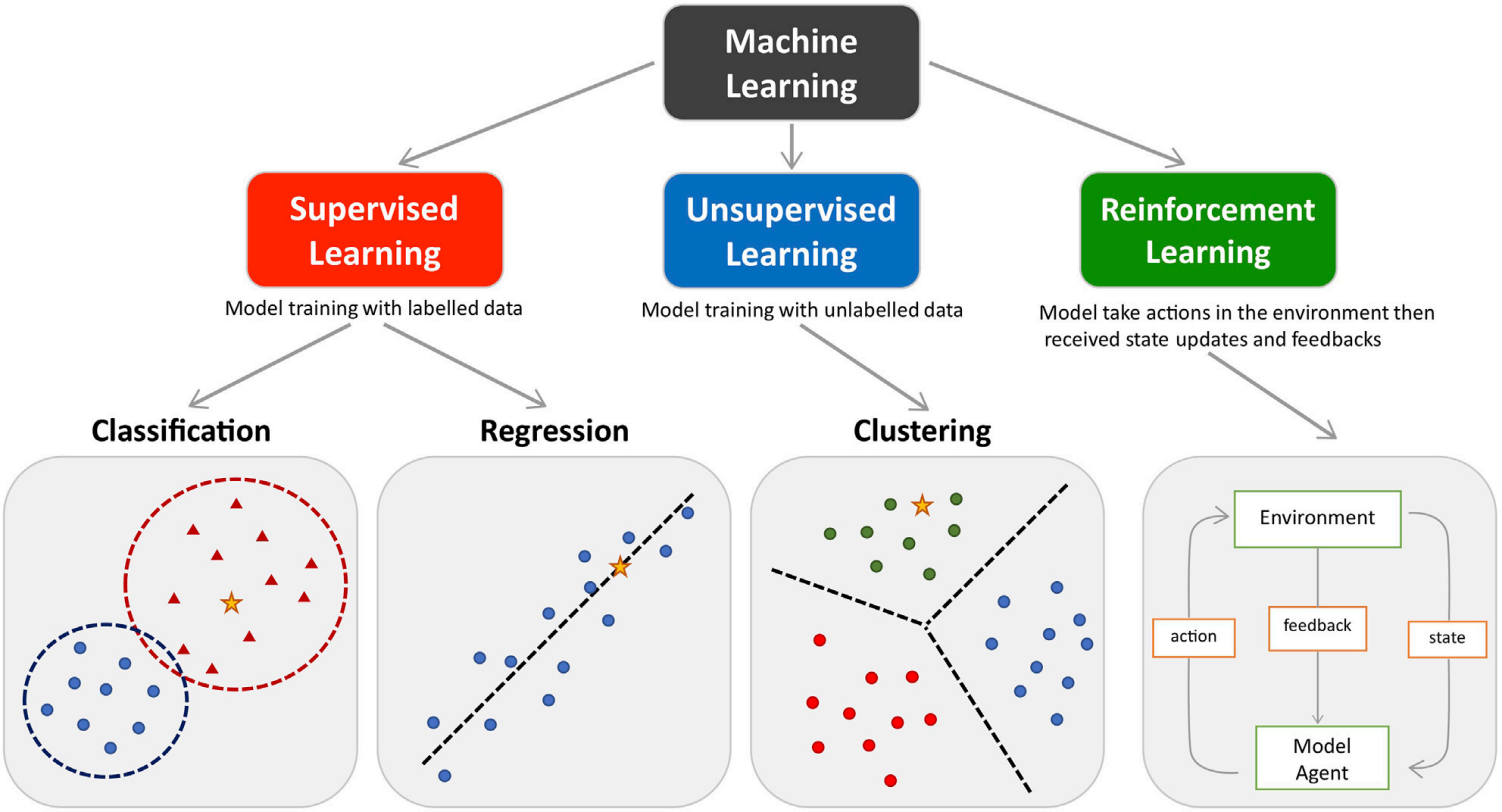
The main types of machine learning. Main approaches include classification and regression under the supervised learning and clustering under the unsupervised learning. Reinforcement learning enhance the model performance by interacting with environment. Coloured dots and triangles represent the training data. Yellow stars represent the new data which can be predicted by the trained model.

ML approaches can be generally divided into three types: supervised, unsupervised and reinforcement learning, tailored for distinct investigation purposes (Figure 1 and *Glossary*). Supervised learning algorithms investigate relationships between predictive variables and outcome from labelled training datasets and apply the learned rule to establish a model for classifying new data (Russell et al., 2010). Classification and regression are two major approaches in supervised learning, where the classification model aims to predict category outcome (e.g., diagnosis given by clinician) and the regression model aims to predict a continuous outcome (e.g., disease activity score). The application of supervised learning models is crucial for biomarker identification in precision diagnostic and therapeutic decision making, as well as predicting disease prognosis. Conversely, unsupervised learning algorithms are applied to uncover hidden patterns in training data without labels. Clustering approaches within unsupervised learning, including hierarchical clustering, K-means clustering and Gaussian mixture models, are the most popular techniques for assembling data into previously ambiguous bundles. Unsupervised clustering approaches form the decisive component in most patient stratification studies and in identifying disease subtypes (Mossotto et al., 2017; Orange et al., 2018; Robinson et al., 2020; Martin-Gutierrez et al., 2021). Finally, reinforcement learning is scripted to sequentially self-correct from environmental feedback (positive or negative) and therefore improve the overall model function without having labelled data (Kaelbling et al., 1996). While the application of reinforcement learning is less prevalent in clinical research compared to supervised and unsupervised learning, the value of reinforcement learning in clinical trial design is highlighted in numerous studies (Padmanabhan et al., 2015; Yauney and Shah, 2018; Ribba et al., 2020). Moreover, deep learning, inspired by the biological neural communication networks in the brain, is a noteworthy subset of ML algorithms for processing data and extracting patterns that are used for decision-making. Deep learning can be designed as a supervised, unsupervised or reinforcement model, which allows it to handle a variety of tasks. Popular deep learning algorithms such as recurrent neural networks (RNN) and convolutional neural networks (CNN) are powerful tools in the field of computer vision, where medical imaging recognition is widely studied for disease diagnosis (Le et al., 2009), prognosis (Klang et al., 2020) and subtypes identification (Suzuki, 2017; Jaber et al., 2020).

### Data Types

The tremendous expansion of patient-derived data accounts for the popularity of ML approaches in the quest for precision medicine. Extensive types of patient data are collected as part of electronic health records (EHR) (e.g., patient demographic data, routine clinical and serological measurements, imaging data) and clinical research (e.g., omics data).

Data characteristics, such as universality and potential applicability for developing effective precision medicine approaches, facilitate ML-based clinical studies. Electronic medical records (EMR) data are the most systematically collected patient data with standardised format and are frequently applied in clinical ML applications because they are relatively accessible and easy-to-implement. EMRs are digital data compiled by healthcare systems, they contain longitudinal information from individuals, such as medical history, current diagnoses, medication, disease activity and other clinical measurements collected at a particular clinical visit. EHRs contain information beyond EMRs, including cumulative laboratory and imaging data available for a certain patient as well as information about their overall health from all the clinicians involved in their care. Applying ML to data from EMR/EHRs is a major area of interest within the field of personalised diagnosis and treatment (Landi et al., 2020).

Medical imaging including magnetic resonance imaging (MRI), computed tomography, nuclear imaging, x-ray, electroencephalography and ultrasound etc., are all techniques with standardised imaging acquisition protocols. These data are predominantly analysed by deep learning algorithms, which are the most suitable due to their strength and competence in analysing the complex detail present in medical images. Deep learning techniques have shown particular progress in precision oncology including early diagnosis, identifying cancer subtypes, early detection of metastasis and aiding clinical decision-making (Liu et al., 2019; Munir et al., 2019; Tandel et al., 2019).

There are various applications of ML techniques in radiology, from automatization of routine tasks usually performed by radiologists and clinicians requesting various investigations, such as assessment of imaging appropriateness, creating study protocols to improve image quality and minimise radiation, and standardisation of the way radiology studies are reported (Lakhani et al., 2018).

Although the majority of ML applications in radiology are not specific for use in immune-mediated chronic inflammatory conditions, which are the focus of our review, various ML algorithms have been implemented in clinical practice, such as *medical image segmentation* (Cooper et al., 1998) which can be applied to various types of imaging (e.g., brain, spine, lung, liver, kidney, colon); *medical image registration* (e.g. integration of various complementary imaging modalities or time series to facilitate diagnosis); *computer-aided detection and diagnosis* (Doi, 2007) (e.g., mammography, CT colonography, and CT lung for detection of nodules which assist clinicians in diagnosis by reducing reading time and improving the sensitivity of the detection of pathological findings); *brain function/activity analysis and diagnosis of neurological conditions using functional MR (fMR) images* (Pereira et al., 2009) (to facilitate the non-invasive interpretation of high dimensional data related to the brain function); *content based image retrieval* systems which enables searching for digital images in large databases based on the contents of the image to facilitate diagnosis by comparing images with similar features or from previously-confirmed cases with the same diagnosis; and *text analysis of radiology reports* (Dreyer et al., 2005) using natural language processing (NLP) and natural language understanding (NLU) (Wang and Summers, 2012).

Biomarker discovery and application is a main focus in modern-day clinical research, where quantified molecular signatures are used as indicators for predicting different aspects of certain diseases. Compared to traditional evaluation of patients by direct clinical observations of the disease presentation, multiple biomarker panels from high dimensional data measured by state-of-the-art technology allow researchers to pinpoint disease endotypes from a wide spectrum of clinical presentations and could be particularly important for precision medicine in complex human diseases. For disease diagnosis, biomarkers that can be routinely collected by cheap and easily accessible approaches are preferable since periodic assessment is crucial for disease detection and early intervention of high-risk populations. Alternatively, prognostic biomarkers for predicting associations with mortality, disease progression, and more active disease, usually involve disease specific investigations, including analysis of blood (Robinson et al., 2020; Coelewij et al., 2021), urine (Glazyrin et al., 2020), cerebrospinal fluid (Toscano and Patti, 2021), tears (Torok et al., 2013) and even breath (Sola Martínez et al., 2020), as well as routinely collected imaging data (Ciurtin et al., 2019). Omics analysis of such biological material, including metabolomics, proteomics, RNA-sequencing (so-called “big data”) and autoantibody data are used to study diagnosis and prediction of disease activity in inflammatory chronic diseases (Teruel et al., 2017; Imhann et al., 2019). Furthermore, digital clinical data extracted from EHR can potentially provide digital biomarkers for disease diagnosis and risk prediction (Wu et al., 2017). With the power of deep learning, biomarkers extracted from imaging data have already extended the accuracy of human decision-making (Liu et al., 2019). However, the expensive operating cost, the invasiveness of certain imaging approaches and the demand of a relatively large data size to generate meaningful outcomes from ML models are major drawbacks for applying imaging biomarkers in ML-based clinical research. For predicting treatment response such as treatment resistance and recurrence risk in inflammatory diseases, genetic, serological and immunological biomarkers and clinical phenotyping are frequently applied (Bek et al., 2016; Figgett et al., 2019; Waddington et al., 2020).

### Workflow for Building Machine Learning Models

**FIGURE 2:**
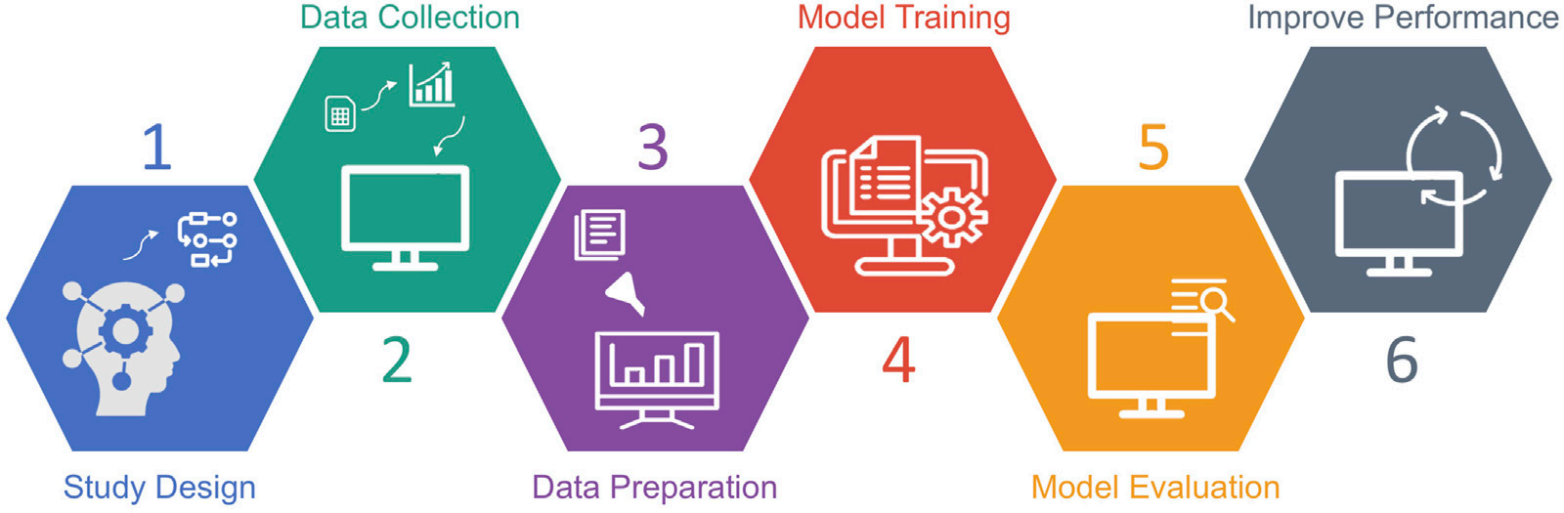
Workflow of machine learning study.

To be intelligent and provide new solutions for intractable clinical needs, ML needs to learn and improve from the given data and apply it in a dynamic environment. Essential steps involved in building a ML model include study design, data collection, data preparation, model training, model evaluation and performance improvement (Figure 2). Before the actual model training, a thoughtful study design that answers key questions including what the unmet clinical need is, what types of data need to be collected and applied, what types of ML are suitable to address the study aims etc., are critical for building effective ML models with suitable clinical value. Gathering data is the first and most important step of any ML approach, since making inferences from a given sample is the core task of ML. The quantity and quality of the collected samples determine whether the model is effective and representative when applied in practice. Subsequently, the data preparation process prunes the raw data into a specific format. Models are constructed using the training dataset and further evaluated using the validation/testing dataset. The model validation includes internal validation (e.g., k-fold cross-validation) and external validation using an external cohort. Finally, model performance is enhanced by repeatedly undergoing model training and evaluation processes until the performance is optimal.

## Applications

### Machine Learning Applications in Immune Mediated Inflammatory Disease: Prediction, Diagnosis and Prognosis

One of the main strengths of ML is the ability to analyse data with many variables and perform biomarker selection, which could contribute to precision diagnosis and prognosis. Traditional analysis techniques tend to examine linear relationships between individual variables and outcomes and are often heavily dependent on existing knowledge, which is often inefficient and short-sighted when dealing with datasets with overwhelmingly high dimensions, as is the case with omics data. In contrast, ML approaches can sufficiently handle a large number of variables in the dataset and can also quantify and rank the variable importance in model training. For example, the “mean decrease in Gini” in the random forest model measures the average (mean) of the total decrease in node impurity of variable, weighted by the proportion of samples reaching that node in each individual decision tree in the random forest; thus, a higher “mean decrease in Gini” implies a greater contribution of a variable to the overall model performance (see *Glossary*). ML methods allow a robust biomarker selection process, enabling researchers to quickly screen out and combine the most relevant markers for more comprehensive decision-making. Effective biomarker selection has been applied extensively in diseases with a strong genetic determinant such as cancer (Henry and Hayes, 2012). However, this is more challenging in multifactorial diseases with substantial environmental susceptibility factors such as autoimmune inflammatory diseases.

#### Machine Learning for Diagnosis

There are multiple examples in the literature where predictive ML models have been used to identify diagnostic biomarkers in immune mediated inflammatory diseases (Table 1) (Seyed Tabib et al., 2020; Stafford et al., 2020). For example, ML techniques applied to proteomics have differentiated between immune-mediated CKD and other causes of CKD (Glazyrin et al., 2020). In this study, plasma proteomics data from 131 subjects balanced across CKD disease patient subtypes (diabetic nephropathy, glomerulonephritis and hypertensive nephropathy) and healthy controls were analysed. Principle component analysis (PCA) selected 175 relevant protein predictors, which were individually assessed using conventional statistical methods, but no significant differences were identified between the groups. However, using the K-nearest neighbours ML model, the CKD disease group was discriminated from the healthy group with a 97.8% accuracy, and patients with diabetic nephropathy were separated from glomerulonephritis patients with a classification accuracy of over 96%. A similar approach was performed with proteomic analysis of 47 urine samples, which separated healthy controls from CKD disease with high performance but failed to effectively discriminate within CKD disease subtypes (Glazyrin et al., 2020). However, the extremely small dataset in the urine study (eight samples in the smallest group) greatly limited the power of the ML model as well as giving an unreliable model performance, due to the concern of model overfitting the training data (to be discussed in a later section). Although many ML approaches can deal with the classification of multiple groups, a decrease in robustness for most models is inevitable when the number of classes increases. To overcome this, the above study proposed a two-stage differential diagnosis; the urine-based ML model for separating hypertensive nephropathy and healthy control samples from patients with CKD, to be followed by the plasma-based model to separate patients with glomerulonephritis and diabetic nephropathy. Thus, this study provides a potential early diagnosis strategy using proteomics-based ML-models coupled with the ability to differentiate between disease subtypes. This could decrease the use of invasive kidney biopsies, although further external validation on a large cohort is essential.

**TABLE 1:** Examples of machine learning application in precision diagnosis and prognosis of inflammatory diseases.

ML algorithms	Type of data	Sample sizes	Applications	References
Applications in Disease Diagnosis				
kNN, LR, SVM, DT, PCA	Plasma and urine proteomics	131 plasma and 47 urine samples from CKD patients	Proteomics-based ML approach was developed as differential diagnosis tool of early state CKD.	Glazyrin et al. (2020)
RF	Immunophenotyping	72 JIA and 43 healthy controls	ML methods applied to identify JIA patients from healthy controls by immune profile	Van Nieuwenhove et al. (2019)
SVM, RF, kNN, NB	fMRI connectivity matrix	41 neuropsychiatric SLE patients and 31 healthy controls	ML classifiers applied for Neuropsychiatric SLE patients using resting-state fMRI functional connectivity	Simos et al. (2019)
unsupervised surrogate assisted feature selection (SAFE), NLP, LR	Electronic Health Records	114 definite SLE, 49 probable SLE, 237 Non-SLE patients	ML algorithms were applied to identify lupus patients in electronic health records and validated the performance of existing rule-based algorithms	Jorge et al. (2019)
AdaBoost	Electronic Health Records	583 SLE, 16174 non-SLE patients	ML model trained with noisy labelled electronic health records are used for heterogenous lupus identification	Murray et al. (2018)
Applications in Disease Prognosis				
Elastic generalized linear model (GLM), KNN, RF	Whole blood gene expression data	156 SLE (82 active; 74 inactive) patients	Supervised ML approaches were applied to predict lupus disease activity using gene expression data	Kegerreis et al. (2019)
Multinomial LR	Laboratory measurements and demographics	286 SLE with 5,680 visits	Screening ML models to identify high disease activity SLE patients using simple demographic and laboratory measurements	Hoi et al. (2021)
RNNs	Clinical and laboratory measurements	132 SLE patients with no baseline chronic damage (in the 2 years follow up, 38/132 developed chronic damage)	ML algorithms were used to predict the risk of chronic damage of SLE patients using longitudinal clinical and laboratory measurements	Ceccarelli et al. (2017)
RF, SVM, KNN, AdaBoost, RNNs	Clinical records	1,624 MS patients (follow up visits in 180, 360 and 720 days)	Supervised ML algorithms were applied to predict disease course of MS patients using longitudinal clinical records	Seccia et al. (2020)
Elastic net (GLM)	Quantitative measurements from routine clinical tests	3,515 young and asymptomatic individuals	General linear model was applied to predict subclinical atherosclerosis risk in young and asymptomatic individuals using longitudinal quantitative laboratory measurements and routine clinical tests	Sánchez-Cabo et al. (2020)
RF, LR with and without interaction, SVM, DT	Serum metabolomics data	80 female SLE patients	Supervised ML classifiers were applied to predict subclinical atherosclerosis in SLE patients using serum metabolomics data	Coelewij et al. (2021)

Abbreviation: ML, Machine learning; PCA, principal component analysis; LR, logistic regression model; GLM, generalized linear model; SVM, support vector machine; GB, gradient boosting; XGBoost, extreme gradient boosting; RF, random forest; DT, decision tree; ET, extremely random trees; GBDT, gradient boosting decision tree; NB, naïve Bayes; NN, neural network; CNN, convolutional neural networks; RNNs, recurrent neural networks; DL, deep learning; kNN, k-nearest neighbours; NLP, natural language processing; CKD, chronic kidney disease; JIA, juvenile idiopathic arthritis; SLE, systemic lupus erythematosus; MS, multiple sclerosis.

Researchers have also explored precision diagnosis of juvenile idiopathic arthritis (JIA), a heterogeneous autoimmune disease, using immune-based ML approaches (Van Nieuwenhove et al., 2019). Immunophenotyping data of 72 JIA patients and 43 age-matched healthy controls were used as predictors for the classification model (random forest). After optimisation and 10-fold cross-validation, the random forest model had high performance with an area under the curve (AUC) of 0.90 when discriminating JIA from healthy using all 42 immune cell subtypes. iNKT cell subtype was the variable that contributed most to the random forest model (assessed by mean decrease in Gini), and was used to build a univariable (iNKT cell only) model which had an AUC of 0.91. However, after removing iNKT cells from the model (keeping all other predictors), the model maintained a good performance (AUC = 0.86). The order of the variable ranking also remained the same in models with and without iNKT cells. These results suggested that the contribution of iNKT cells to JIA pathogenesis may not be the most important despite being the top ranked variable by the random forest model. The study illustrates the power of ML analysis in explaining biological function and the potential clinical application in precision diagnosis of JIA.

In a study of patients with neuropsychiatric SLE (Simos et al., 2019), researchers applied a ML model to enhance current neuropsychiatric SLE diagnosis approaches based on resting-state functional connectivity MRI (fMRI) imaging data of the brain. ML classifiers, including random forest, support vector machine, naïve Bayes and k-nearest neighbours were trained by the fMRI connectivity matrix derived from fMRI images of the brain network of 41 neuropsychiatric SLE patients and 31 healthy controls. The support vector machine model achieved the best performance, identifying neuropsychiatric SLE patients with and AUC 0.75, validated by 5-fold cross-validation. This model also indicated that the frontoparietal brain region contributed most to the performance. However, the model performance is not outstanding for practical use in diagnosis, therefore testing a larger cohort for model training and performing appropriate external validation in future studies could potentially elevate the model quality and help build a neuropsychiatric SLE classification pipeline.

A number of studies have begun to examine the application of ML techniques to the diagnosis of complex autoimmune diseases using EHR and EMR data (Murray et al., 2018; Jorge et al., 2019). In a previous study by Jorge et al. (2019), ML algorithms were able to identify patients with systemic lupus erythematosus (SLE), a complex disease whose diagnosis requires multiple criteria, including clinical presentation, history of symptoms and, laboratory data. Patients with an international classification of disease (ICD) code that suggested a possible diagnosis of SLE (without fulfilling the criteria for classification as having SLE) were included in the model training. Selected EMR records were then defined, and the corresponding patients were assessed by rheumatologists using clinical expertise and validated SLE classification criteria, and categorised as either definite SLE, probable SLE and non-SLE. A novel ML approach combined the rule-based and natural language processing (NLP) algorithms (Teller, 2000) to identify SLE patients using EHR data (including laboratory measurements, medications and disease history). The model achieved an overall good performance (AUC = 0.909) with a 92% positive prediction rate when classifying SLE (definite and probable) from non-SLE cases. Although the performance of ML models was not improved compared to the rule-based methods, the combined method demonstrated a good performance on both internal and external validation. This is particularly important for developing a portable and universal pipeline for identifying SLE patients based on medical records and implementing into a healthcare system and could provide a model for classifying complex diseases such as SLE.

In another study using EHR data to identify SLE patients (Murray et al., 2018), an ensemble algorithm (AdaBoost learners, EasyEnsemble (Liu et al., 2009)) was applied to an imbalanced dataset (derived from 583 SLE, and 16174 non-SLE individual patient EHR). A high model performance was achieved (AUC 0.97) and maintained in the testing dataset (AUC 0.94), where definitions of SLE were validated by two rheumatologists using “strict” and “inclusive” terms respectively.

Similar studies have applied EMR data to classify patients with rheumatoid arthritis (RA) (Liao et al., 2010) and IBD (Ananthakrishnan et al., 2013), as well as to identify patient subsets. For example, a study used EHR to identify methotrexate-induced liver toxicity in RA patients (Lin et al., 2015). A logistic regression model was used to classify cases as having or not methotrexate induced liver toxicity, with a 0.756 positive predictive value. Moreover, EHR-based ML models can be used to screen for genetic disorders with long term health effects such as familial hypercholesterolemia, which can remain largely undiagnosed due to the strict privacy rules for universal screening in some areas. A “random forest” -based ML algorithm (FIND FH) developed by Myers and colleagues (Myers et al., 2019) identified individuals with a high chance of having familial hypercholesterolemia using information available on external healthcare system databases. Samples from the identified individuals at risk for FH were further validated by experts with a precision ranging from 77 to 87%, showing that EHR-based ML models could be a promising preselection tool for identifying patients at risk for genetic conditions without universal screening.

#### Machine Learning in Predicting Disease Prognosis

ML classification models can also be applied in disease activity prediction of complex autoimmune diseases (Table 1). This has been attempted in several ways as can be demonstrated in SLE. In a study using whole blood gene expression data, SLE disease activity was predicted by ML classifiers (Kegerreis et al., 2019). The gene expression and module enrichment data of 156 SLE patients from three datasets were included and stratified for disease activity using the Systemic Lupus Erythematosus Disease Activity Index (SLEDAI); active disease SLEDAI≥6 and inactive disease SLEDAI<6. Interestingly, both conventional gene differential expression analysis and unsupervised clustering methods (hierarchical clustering) failed to distinguish SLE patients based on their disease activity alone, potentially due to the heterogeneous and complex nature of the disease. Therefore, supervised ML classifiers including random forest, k-nearest neighbours and generalized linear models were used to separate patients with active versus inactive disease. The random forest classifier scored the highest performance with a peak accuracy of 83% when using raw gene expression data as a predictor. However, the model performance varied dramatically when validated by datasets using different technical settings. When gene modules were used as model predictors, the performance of the random forest classifier was stable at around 70% accuracy regardless of the validation approach. The mean decrease in Gini impurity from the random forest model indicated an important role for CD14^+^ monocytes in SLE patients with active disease. Although models trained by gene expression data remain challenging for implementation in the clinical setting from the point of view of feasibility and cost-effectiveness, the gene expression features identified between active and inactive groups of patients may boost the understanding of SLE pathogenesis.

Another study attempted to identify SLE patients with high disease activity using ML algorithms without making use of the validated disease activity score usually implemented in routine practice (SLEDAI) (Hoi et al., 2021). The longitudinal data of 286 SLE patients (median follow up 5.1 years, a total of 5,680 visits) including measurements of High Disease Activity (HDA), defined as SLEDAI-2K≥10, 16 laboratory and three demographic parameters (age, sex, and ethnicity) were used to build a multinomial logistic regression model. After screening a total of 2^16^ models with different variable settings for optimisation purposes, the final model including seven laboratory variables and three demographic variables identified with 88.6% accuracy whether a certain SLE patient had HDA or not. The model training used data from all visits, irrespective of their time point and this limited the possibility of using certain earlier time-points to predict later disease development status. The study shows the possibility of using a limited amount of routinely available laboratory measurements and demographics to select SLE patients with HDA, which could help the early identification of SLE patients likely to require treatment escalation after testing the model in a clinical setting.

Another study accurately predicted chronic damage in SLE with the aim to improve disease management (Ceccarelli et al., 2017). 413 SLE patients were assessed for chronic damage evaluated by the validated SLICC/ACR Damage Index (SDI) (Gladman et al., 1996), which includes longitudinal measurements of damage potentially acquired within 12 organ systems. Supervised recurrent neural network (RNN) which is a class of artificial neural network (see *Glossary*) was employed to classify patients without chronic damage at baseline but who developed damage in the following 2 years versus those who did not develop chronic damage. Clinical data including demographics, diagnosis date, co-morbidities and medical history, and laboratory data including important markers of SLE were used as predictors for RNN model training. The RNN model uses the all the longitudinal time point (≥5 visits for each patient) of chronic damage measurement as the sequential input, then processes the network through the hidden layer (layers in between) until connecting the output layer, which generates the prediction results (see *Glossary*). To avoid overfitting, an early stopping technique (stop when AUC reaches 0.95) and 8-fold cross-validation were applied. The model performance was stable at AUC (0.77) for predicting a chronic damage-developing group.

Similar studies have also been described in patients with MS. Seccia and colleagues applied supervised ML algorithms to predict disease progression of MS and potentially provide treatment decision support (Seccia et al., 2020). Four common ML algorithms (random forest, support vector machine, k-nearest neighbours and AdaBoost) were employed to identify whether patients with MS will evolve from the initial Relapsing-Remitting (RR) phase to the Secondary Progressive (SP) phase over 180, 360 and 720 days using real-world clinical data. After model optimisation, the prediction accuracy of random forest, support vector machine, and AdaBoost models had similar performances around 85% for 180-, 360- and 720 days progression prediction. Due to the nature of MS evolution, the sample size of transitioning (SP) patients is usually significantly smaller than the non-transitioning (RR) patients. This extremely imbalanced data limited the overall performance of the model and could be improved by a larger study cohort with more balanced data and external validation. Moreover, a more integrated and comprehensive approach combing results from all the high performing models could improve the overall prediction.

The classification and biomarker selection properties of ML algorithms can also help to predict the prognosis of diseases with a long asymptomatic phase. In a recent study of atherosclerosis, Sánchez-Cabo and colleagues applied ML to predict cardiovascular risk in asymptomatic individuals (Sánchez-Cabo et al., 2020). Non-invasive imaging such as computerised tomography and vascular ultrasound can help to assess cardiovascular risk but are only recommended in clinical practice after evaluating traditional risk factors such as serum cholesterol levels, which could underestimate the long-term cardiovascular risk in asymptomatic individuals. In this study, ML models were built based on 3,515 individuals with 115 quantitative predictors collected from routine clinical tests. Baseline imaging was used to classify samples into four groups (no disease, focal disease, intermediate disease, generalized disease) based on the detection of subclinical atherosclerosis. The “no disease” and “generalized disease” classes were used to build up an elastic net model (penalized linear regression model) (see *Glossary*) using all predictors. After variable selection from the model, a refined model with 12 predictors was employed. The refined elastic net model significantly outperformed the traditional cardiovascular risk assessment scores in predicting generalized subclinical atherosclerosis and the risk of progression in 3 years. Notably, this model improved the false-negative prediction rate meaning that fewer high-risk individuals were mis-classified in the “no disease” group.

In a recent study of SLE (Coelewij et al., 2021), researchers attempted to predict subclinical atherosclerosis in SLE patients using serum metabolomics data. 228 metabolites from 80 female SLE patients were quantified by nuclear magnetic resonance spectroscopy and used as predictors. Subclinical atherosclerosis status of each patient was assessed by femoral and carotid artery ultrasound scans. After pre-processing the serum metabolomics data (imputation of missing data, homology reduction and data scaling), five supervised classification models were applied to predict subclinical atherosclerosis. The logistic regression with interactions model achieved the highest classification accuracy (80%). Feature selection was performed using the top three models (random forest, logistic regression with and without interaction) in predicting subclinical atherosclerosis in SLE, where very low-density lipoprotein (VLDL) subclasses and leucine were top ranked in the ML model and were also validated by the univariate logistic regression. As SLE patients are known to be at higher risk of developing cardiovascular disease compared to age and sex-matched healthy individuals, this study revealed the possibility of using serum biomarkers to identify SLE patients with high cardiovascular disease risk early and allow adequate preventative strategies to address this risk. ML techniques have also been used for complex risk disease prediction using both genetic and nongenetic data with different levels of performance. A 7 years longitudinal study in patients with hepatitis C identified that boosted-survival-tree models were statistically superior to cross-sectional or linear models for predicting development of cirrhosis in chronic hepatitis C as a model of a disease with a non-linear progression trajectory (Konerman et al., 2019). However, a benchmarked polygenic risk score which did not account for possible nonlinear effects, had a better prediction capacity for coronary artery disease than various ML techniques, such as penalized logistic regression, naïve Bayes, random forests, support vector machines, and gradient boosting when tested on an independent data set (Gola et al., 2020). This suggests that although overall ML strategies can improve the predictive capacity of individual or composite biomarkers commonly used in research or clinical practice, the added value of ML heavily depends on the quality and the relevance of the data fed into the model.

#### Machine Learning for Disease Subtype Identification and Therapy Selection

Personalised treatment is a fundamental aim of precision medicine, where individuals receive tailored therapy instead of the one-size-fits-all approach. The precision of the treatment is increasingly important in heterogeneous diseases, including autoimmune inflammatory diseases, where significant disease signature differences between patients can be overlooked by the same diagnosis. An effective way of delivering personalised treatment is by performing a more precise subpopulation identification based on their distinct pathogenetic signatures. Signatures can be extracted from genomes, metabolomics, immunophenotyping and other types of data. Supervised ML is an ideal tool, specialised in the identification of unique signatures, while clustering approaches from both supervised and unsupervised ML are designed for partitioning complex high dimensional data. An increasing number of studies have applied ML models to identify subgroups of patients and show promising results toward more personalised treatment (Table 2) (McKinney et al., 2010; Waljee et al., 2019; Mo et al., 2020; Rehberg et al., 2020).

**TABLE 2:** Examples of machine learning application in subtype identification, therapy selection and drug development of inflammatory diseases.

ML algorithms	Types of data	Sample sizes	Application	References
Applications in Disease Subtype Identification and Therapy Selection				
PCA, PLSDA, sPLS-DA, k-means clustering, hierarchical clustering	Whole-blood RNA sequencing data	161 SLE and 57 healthy controls	ML clustering approaches were applied to stratify SLE patients based on gene expression signatures	Figgett et al. (2019)
RF, sPLS-DA, k-means clustering	Immunophenotyping	45 SS, 29 SLE,14 patients with both conditions and 31 healthy controls	ML and statistical approaches were applied to discover shared immune profile between SS and SLE. Immune cell signatures were used to stratify patients into groups with different clinical presentation regardless of the diagnosis	Martin-Gutierrez et al. (2021)
RF, sPLS-DA, k-means clustering	Immunophenotyping	67 juvenile-onset SLE patients and 39 healthy controls	ML and statistical approaches were applied to identify juvenile-onset SLE from healthy controls using immunophenotyping data. The immune cell signatures were used to stratify patients into four groups with different clinical manifestations	Robinson et al. (2020)
XGBoost, RF, GBDT, ET and LR	Electronic Medical Record	87 JIA patients with etanercept treatment	Supervised classifiers were applied to predict the treatment efficacy of etanercept in JIA patients	Mo et al. (2020)
DT, RF, kNN, SVM, LR with and without interactions	Serum metabolites	89 MS patients with IFNβ treatment	Supervised classifiers were applied to predict the anti-drug antibody development in MS patients before and after IFNβ treatment	Waddington et al. (2020)
Applications in Drug Development				
DL (deepDTnet)	15 types of chemical, genomic, phenotypic, and cellular network profiles	732 small molecules	A DL approach was developed for novel target identification and drug repurposing using heterogeneous drug–gene–disease networks from existing drugs	Zeng et al. (2020)
Bayesian network (BANDIT)	Drug efficacies, post-treatment transcriptional responses, drug structures, reported adverse effects, bioassay results and known targets	>2,000 small molecules	A Bayesian machine learning approach was developed for novel binding target prediction using diverse data types	Madhukar et al. (2019)
Translational Network for Indication Prediction (CATNIP)	16 different drug similarity features	2,576 small molecules	ML algorithm was developed for drug repurposing using only biological and chemical information of the molecules	Gilvary et al. (2020)
DL (MathDL)	Public databases (PDBbind and ChEMBL)	17,382 protein–ligand complexes (PDBbind) and 2 million compounds (ChEMBL)	DL and algebraic topology were used to rank the attractive binding sites for SARS-CoV-2 drug development. The model identified 71 covalent bonding inhibitors for SARS-CoV-2 main protease, a favourable drug target of SARS-CoV-2	Nguyen et al. (2020)

Abbreviation: ML, Machine learning; PCA, principal component analysis; sPLS-DA, sparse partial least squares-discriminant analysis; LR, logistic regression model; SVM, support vector machine; GB, gradient boosting; XGBoost, extreme gradient boosting; RF, random forest; DT, decision tree; ET, extremely random trees; GBDT, gradient boosting decision tree; NB, naïve Bayes; NN, neural network; CNN, convolutional neural networks; RNNs, recurrent neural networks; DL, deep learning; kNN, k-nearest neighbours; NLP, natural language processing; SLE, systemic lupus erythematosus; SS, Sjögren’s syndrome; JIA, juvenile idiopathic arthritis; MS, multiple sclerosis; SARS-CoV-2, severe acute respiratory syndrome coronavirus 2.

SLE is a chronic ARD with no cure. Due to the heterogeneous nature of SLE, predicting treatment response of SLE patients remains challenging. Figgett and colleagues (Figgett et al., 2019) applied ML clustering approaches to perform SLE patient stratification using whole-blood RNA-sequencing data. Both unsupervised clustering (PCA, k-means clustering) and supervised clustering (partial least squares-discriminant analysis, PLS-DA) approaches were applied to the gene expression data from 161 SLE and 57 healthy samples. Unsupervised PCA provided an overall view of the gene expression data, which confirmed a higher heterogeneity in SLE compared with healthy controls. On the other hand, supervised PLS-DA maximised the difference between SLE and healthy controls with the help of labelled data, and selected top-weighted genes from the model. The SLE patients were then stratified into four clusters (C1–C4) with different gene expression signatures by k-means clustering. These identified clusters were supported by ML classifiers, where an 88% accuracy of model performance showed a clear divergence between these SLE subpopulations. From the enrichment analysis, C1 had the most similar gene expression architecture to healthy samples. Investigating the clinical manifestations of the clusters identified that flare activity was significantly elevated in C3 and C4; significantly more renal disorder and discoid rash in C4; significantly more serositis in C2. Moreover, using PLS-DA, genes related to disease flare were identified and used to discriminate between flare and non-flare patients, and enrichment analysis of the selected genes identified an increase in inflammatory signalling such as IL-6 and TNF-α, upregulated proliferation signalling, and haematological disturbances. This study improved the understanding of SLE heterogeneity and provides insight for potential personalised treatment in subpopulations of SLE patients.

In the recent study of primary Sjögren’s syndrome (pSS) and SLE (Martin-Gutierrez et al., 2021), researchers applied supervised ML models to identify shared immunological characteristics between pSS and SLE. These two diseases share some clinical and laboratory features, despite differences in disease pathogenesis and overall clinical presentation, leading to a distinct diagnostic label (Pasoto et al., 2019). Immunophenotyping data comprising 29 immune cell subsets from 45 SS, 29 SLE, 14 patients with both conditions and 31 healthy controls was generated by flow cytometry. A range of analysis including supervised ML models (balanced random forest and sparse partial least squares discriminant analysis), univariate logistic regression and multiple t-tests were used to confirm the immunological similarity between pSS and SLE. Thus, all patient’s data was then combined (*n* = 88) and stratified by k-means clustering into two groups with distinct immune profiles. The balanced random forest model identified a signature of eight T-cell subsets that differentiated between the two groups with high performance (AUC = 0.99). The 5 year clinical trajectory analysis identified differential damage scores and disease activity between the two groups. The study suggests the potential of differentiating pSS and SLE patients based on their immunological profile and could provide the opportunity for more accurate targeted treatments across diagnostic boundaries.

ML applications can be used to predict drug efficiency and provide precise treatment support for heterogeneous diseases. In a study of JIA (Mo et al., 2020), ML algorithms were employed to predict the efficiency of biological therapy (etanercept) in JIA patients using EMR data. A wide range of supervised ML approaches including extreme gradient boosting (XGBoost), random forest, gradient boosting decision tree (GBDT), extremely random trees and logistic regression were tested as potential predictive models. EMR data from 87 JIA patients receiving weekly etanercept treatment at the same dose (0.8 mg/kg) were used for model training. The efficacy of the etanercept treatment was assessed using a standard disease activity score validated in adults with RA (DAS44/ESR-3) (Ranganath et al., 2007; Consolaro et al., 2009) at baseline and 3 months after treatment, where a drop in DAS44 >0.6 was considered as a response to treatment. Feature selection was performed in each ML model. After optimisation, XGBoost outperformed the other models with an AUC 0.79 indicating a good predictive performance. Although an external validation was employed, this was small in number (only 14 patients) thereby limiting the reliability of the validation and the ability to apply the model in practice. Another study identified a limited contribution of genetic markers in addition to clinical parameters in predicting response to anti-TNF therapy in RA using a Gaussian process regression model which correctly classified patients’ response in 78% cases (Guan et al., 2019). A recent ML application for personalised treatment response in RA investigated with success molecular signatures predictive of response to adalimumab and etanercept using differential gene expression in peripheral blood mononuclear cells (PBMCs), monocytes and CD4^+^ T cells and methylation analysis in PBMCs (Tao et al., 2021). The random forest algorithms implemented to exploit the transcriptome signatures had an overall accuracy of 85.9 and 79% for response to adalimumab and etanercept and they have been validated in a partial dataset (a follow-up study).

Another study tried to predict anti-drug antibody development in MS patients treated with interferon β (IFNβ) (Waddington et al., 2020). More than one third of MS patients treated with IFNβ develop anti-drug antibodies, which significantly reduces drug efficacy (Bertolotto et al., 2002). Researchers quantified 228 serum metabolites and anti-drug antibody levels of 89 MS patients as part of the ABIRISK consortium (Hässler et al., 2020), at baseline (before treatment), 3 and 12 months after treatment initiation. Six supervised classification models (decision trees, random forest, kNN, SVM, logistic regression with and without interactions) were used to predict anti-drug antibody development (at month 12) and were validated by 10-fold cross validation. The decision tree model outperformed others with a F1 score of 0.788 and a classification accuracy of 0.854 using baseline metabolomics data as predictors. Similar models using serum metabolite levels 3 months after treatment showed better performance in predicting which patients will develop anti-drug antibodies at 12 months by logistic regression models (F1 = 0.88, accuracy = 0.863). The results from variable selection of the models and experimental validation, suggest that serum lipids might play an important role in anti-drug antibody development by changing the lipid composition of immune cell plasma membranes (lipid rafts). Together, this study demonstrates a potential methodology for efficient prediction of drug response using big data (omics and clinical data), which healthcare professionals can use to assess patients earlier for optimal treatment selection.

### Machine Learning for Drug Development

Drug development is a complicated, costly, and time-consuming process, which depends on a large number of factors. The pipelines of drug development can be simply divided into two phases: the drug discovery phase and drug-testing phase (Réda et al., 2020). The drug discovery phase focuses on target identification, target validation and small molecule design, while drug-testing phase includes several preclinical and clinical trials. The complete timeline of drug discovery varies from 5 to 15 years (Réda et al., 2020) with more than 50% failure rate in the late clinical trial phase (Hwang et al., 2016). Due to the high-failure nature of drug development, developing automated approaches with high predictive performance is crucial. To date, numerous studies have investigated the application of ML in drug development (Table 2), aiming to improve the overall success rate by enhancing each step of the drug development process with the extensive use of big data (Vamathevan et al., 2019).

#### Target Identification and Validation

The first step of drug development is target identification which often heavily depends on the extensive study of disease mechanism. Understanding disease mechanisms can be time and labour intensive; common experimental techniques ranging from using immunoprecipitation assays to identify protein-protein interactions in biological samples to genome-wide CRISPR-Cas9 screens to knock down genes of interest. Modern high-throughput techniques generate abundant molecular and biological data, which makes it difficult to screen potential drug targets using conventional methods. To speed up the drug target selection process, numerous studies have developed automated in-silico approaches for drug target identification and validation.

A recent study by Zeng et al. (2020) developed a comprehensive deep learning approach called deepDTnet, which combines networks between drug, gene and disease data to identify novel targets for the existing drugs with great accuracy (AUC = 0.96). Retinoic-acid-receptor-related orphan receptor-gamma-t (ROR-γt) was selected from the deepDtnet approach, as having potential interaction with multiple drugs. An 18-drug screening panel of novel candidates selected from deepDTnet identified that Topotecan (a topoisomerase inhibitor) had an adequate ROR-γt inhibitory capacity (71.0% at 10 μM). Furthermore, this drug was able to ameliorate disease in an experimental mouse model of MS by targeting ROR-γt.

A Bayesian machine learning approach (BANDIT) developed by Madhukar and colleagues (Madhukar et al., 2019) integrated different data types such as treatment response, drug efficacy, molecular structure and adverse effect to predict unknown drug binding targets. The BANDIT model achieved an overall 90% accuracy on more than 2,000 small molecules. By applying the BANDIT approach on 14,000 small molecules with previously unknown targets, novel protein targets for 4,167 small molecules were confidently identified. Furthermore, by applying BANDIT to anti-cancer compounds in clinical development, Dopamine receptor D2 was identified and validated as a target and a compound targeting Dopamine receptor D2 is now undergoing clinical trials for cancer. Overall, BANDIT represents an efficient and accurate platform to accelerate drug discovery and direct clinical application. Together, these approaches overcome the limitation of using only known targets as input data, thus, can discover targets for orphan compounds.

#### Drug Repurposing

Drug repurposing is another powerful application aiming to discover, validate and apply existing approved drugs for new application. The process of drug repurposing is much conserved by renouncing the standard drug development pipeline approach and investigating similarities between various disease processes potentially targeted by the same therapeutic interventions, so that new effective treatments can be delivered faster to patients. This is a more cost-effective approach which led in recent years to the testing and licensing of similar classes of therapeutic agents across many immune-mediated chronic inflammatory diseases (March-Vila et al., 2017; Balasundaram et al., 2019; Gilvary et al., 2020; Martin-Gutierrez et al., 2021). In a recent study of computational drug repurposing, researchers developed a ML (Gradient Boosting model) approach, Creating A Translational Network for Indication Prediction (CATNIP) which can effectively connect similar drugs by solely analysing the biological and chemical data of the molecule without the knowledge of the current therapeutic disease applications of the drug (Gilvary et al., 2020). The CATNIP model was trained with 2,576 small molecules with a good model performance (AUC = 0.84). By performing CATNIP, a strong connection was identified between a kinase inhibitor drug (vandetanib) and diabetes, suggesting that vandetanib could be a potential treatment for type 2 diabetes.

To date, many ML approaches have been applied to discovering effective drugs for acute respiratory syndrome coronavirus 2 (SARS-CoV-2). One of the studies selected the SARS-CoV-2 main protease (M^pro^) as a potential drug target because it was highly conserved and encoded by a distinct gene. Due to the 96.08% similarity between SARS-CoV-2 and SARS-CoV (Xu et al., 2020), researchers hypothesised that inhibitors for SARS-CoV M^pro^ might be effective in blocking SARS-CoV-2 M^pro^ (Nguyen et al., 2020). By combing the mathematics analysis and deep learning models (MathDL), the binding affinity of 137 M^pro^–inhibitors were predicted and ranked without any additional laboratory data. The model revealed that Gly143 was the most attractive residue in M^pro^ and 71 covalent bonding inhibitors interacting with the SARS-CoV-2 M^pro^ were identified. The study extended the current knowledge of the SARS-CoV-2 M^pro^ and provide important information for COVID-19 drug discovery.

Another study applied AI algorithms (BenevolentAI) to explore potential treatment options for COVID-19 using existing anti-cytokine therapies which enabled large-scale clinical trials to be rapidly conducted (Stebbing et al., 2020). Researchers aimed to identify existing drugs that could influence the COVID-19 infection progression by blocking the “cytokine storm” and reduce the associated inflammatory damage associated with a heightened immune response to the virus. Baricitinib is a (JAK)1/JAK2 inhibitor approved for RA treatment which was predicted to have an anti-viral (COVID-19) effect by the BenevolentAI algorithms. The following laboratory validation identified *in-vitro* and *in-vivo* evidence of a reduction in viral infectivity by baricitinib. In a pilot study, four COVID-19 patients were treated with baricitinib resulting in symptom improvement and viral load reduction, providing evidence for clinical benefit derived from ML-driven therapeutic target identification.

## Challenges

Despite the promise of ML research in the field of precision medicine, many challenges still need to be addressed to ensure the further development and acceptance of ML approaches (summarised in Table 3).

**TABLE 3:** Challenges in applying machine learning techniques in precision medicine for immune-mediated chronic inflammatory diseases.

∙ Robust models require sufficient high-quality data	Inadequate sample size in model development can lead to miss representation of the real population and model overfitting. Power calculations under universal guidance are essential during the study design process for ML studies
∙ External validation using independent datasets are an imperative step for predictive model implementation	Lack of external validation is markedly common in studies of autoimmune disease and raises several concerns including model overfitting, poor reproducibility, and generalisability. Online platforms with high-quality and well-defined datasets could enable data reuse which might help researchers with limited access to multiple cohorts to perform model validation
∙ Obstacles in model implementation in clinical practice	Limited interdisciplinary knowledge for translating model metrics to biologically relevant discoveries; lack of usable drugs for model stratified patients; and absence of significant improvement over the traditional approach. These can be improved by using standard practice guidance such as TRIPOD, which allow researchers to carefully assess their model for implementation
∙ Ethical concerns	Clinical predictive models rely on large amounts of personal healthcare data which raise the concern of private data leakage. AI/ML models can discriminate against groups based on ethnicity, gender or economic status due to reliance on biased “real world” data where minority groups maybe underrepresented

### Data Quality

Being a data-driven approach, the performance of the ML model depends heavily on the quality of the data that it builds on. Data needs to have a sufficient sample size and quality in order to represent the target population in the clinical application. In general, a larger sample size is essential for the development of a more robust ML model, which allows accurate prediction for supporting clinical decisions. ML models trained by small sample sizes often suffer from the problem of “overfitting,” where the model over relies on characteristics from the under-represented training data and loses the ability to effectively perform in practice. Similar to the multiple testing issue in conventional statistics, ML models with small sample size might cause false significant discoveries due to random variation under numerous repetitions. For example, one can generate 1,000 different splits of train/test data and evaluate performance. If the performance based on splits shows a great variance, this might indicate an “unstable” model. One way to improve model reliability due to small sample size is by reducing the model variance, as low variance algorithms are less influenced by the specificity of the training data. However, model variance reduction often results in an increase in model biased error, leading to a weakened predictive performance of models (Kohavi and Wolpert, 1996). Meanwhile, obtaining a larger sample size often requires more resources (time, funding, access to large patient populations and computer power etc.). One way to ensure the appropriateness of study design for the research outcome investigated is by having universal guidance of the adequate sample size required for the ML model training for researchers to follow. Studies have already attempted to develop tools to assist decision making in study design. For example, an r package “pmsampsize” was developed to calculate the minimum sample size for the predictive model development to avoid model overfitting, taking into account the number of participants, outcome events and predictive variables (Riley et al., 2019).

However, the use of a limited sample size can be sometimes inevitable due to the rare nature of certain diseases. To overcome the limitation of small sample size, more comprehensive procedures and careful considerations are necessary for generating reliable results. One example is juvenile-onset SLE (JSLE) – a rare ARD. In one study, researchers applied a ML model to stratify JSLE patients based on their immune profile (Robinson et al., 2020). Only 67 JSLE patients and 39 healthy controls with 28 immune cell predictors were included in the analysis. A random forest algorithm was selected as it was less likely to overfit the data due to an implanted bagging method and random feature selection in the model ensembled by a large number of decision trees (Tin Kam, 1995; Breiman, 2001). The results of this model were combined with additional analysis such as the sparse-PLS-DA and univariate logistic regression and were further validated by 10-fold cross-validation. Although the lack of an external validation dataset meant there was still risks for overfitting and not being able to extrapolate the results, the study shows the potential for applying a ML-based pipeline to other rare and heterogeneous immune-mediated inflammatory conditions (Choi and Ma, 2020).

Another challenge in the development of ML models is access to high quality and well-defined datasets, needed for algorithm training and evaluation. In recent years there has been a big push to make research data FAIR (Findable, Accessible, Interoperable and Reusable) (Wilkinson et al., 2016). Datasets generated in research studies should collect enough machine-readable metadata to allow for discovery and searches. Ideally, clear rules for data access and use should be available, as well as use of domain-specific ontologies to describe the data. There should also be enough information available describing how the acquisition of data was carried out, enabling re-use of data.

### Reproducibility and External Validation in Machine Learning

Issues with multiple testing and p-hacking has contributed largely to the reproducibility “crisis” in science. The 2016 Nature survey pointed out that more that 70% of scientists have failed to reproduce other scientists reported results (Baker, 2016). P-hacking in traditional statistics usually means that tests are done on data in an exploratory manner, if something significant is found, a hypothesis is formed based on this finding, i.e., working backwards from data to find patterns and relationships. However, the statistical tests are only valid if the hypothesis is formed first. In ML, working backwards from data to reveal patterns is exactly what is done. In the case of ML, overfitting can be considered the analogy to p-hacking. Overfitting usually means that the ML model can perfectly reproduce training data, but fails on independent data. The way to handle this by data scientists is appropriate internal and external validation of models.

To achieve the highest model performance, many clinical studies tend to avoid data splitting for model development. Resampling methods such as bootstrapping and k-fold cross-validation are economical internal validation, therefore, they are often applied to prevent model overfitting. On the other hand, external validation using an independent cohort is not often performed, potentially due to limited access to similar cohorts, despite being the most straightforward way to evaluate the generalizability of the model. Less than 10% of autoimmune studies combine cross-validation with an independent test dataset for validating model performance (Stafford et al., 2020). However, external validation remains a crucial step for model implementation in real-world clinical practice and the absence of external validation will raise several concerns for the model integrity including bias of the model, lack of reproducibility and lack of model generalizability (Ho et al., 2020). One example is the publication of GWAS studies that are required to have at least two independent data sets for validation to assure a creditable result (Oetting et al., 2017). As external validation requires data from independent sources, access to publicly available online datasets from different studies has become a suitable solution to overcome the lack of independent validation cohorts (Riley et al., 2016). These online databases provide a great opportunity to improve the research quality of ML applications in immune-mediated inflammatory diseases that are often rare conditions associated with a limited number of datasets available. They provide options for researchers to validate their models on more relevant populations, as most current external validation studies use small local datasets simply because of the better accessibility.

### Model Implementation in Clinical Practice

Transforming a well-performed model into an actual clinical application associated with improvement in patient outcomes can be challenging; the term “AI Chasm” describes the discrepancy between the model development and translation of models to real-world applications (Keane and Topol, 2018). The clinical impact of potentially promising ML models requires careful evaluation before considering implementation in clinical settings. For example, a wide range of performance metrics (accuracy, AUC, precision, sensitivity, specificity etc.) (see *Glossary*) are applied to represent the predictive efficacy of ML models in clinical studies. However, most of the metrics do not directly affirm the clinical applicability and can be difficult to evaluate with limited interdisciplinary knowledge (Saito and Rehmsmeier, 2015; Shah et al., 2019). Another common obstacle for the clinical translatability of ML data arrives where emerging ML studies that stratify patients with novel signatures suffer from the lack of effective drugs for the newly identified targets. Furthermore, the reported predictive model needs to provide clinically meaningful advantages over traditional approaches, such as significantly outperform the existing standard statistical approach in relevant fields (Shah et al., 2019). To help address these questions, standard practice guidance is necessary. Reporting of a multivariable prediction model for Individual Prognosis Or Diagnosis (TRIPOD) guideline is an internationally accepted reporting guideline developed to improve the reliability and value of prediction models for diagnostic or prognostic purposes (Moons et al., 2015). TRIPOD-ML focuses on the standardised methodology of ML model development (Collins and Moons, 2019), which together with the interdisciplinary effort from trained experts in different clinical and technology areas of expertise, can ensure that ML applications maximise their chance to translate into precision medicine approaches associated with patient benefit.

### Ethical Concerns

The upsurge of ML applications in personalised medicine has raised potential ethical concerns regarding data privacy, as a wide range of big datasets including personal information from genetics data, demographic data and medication history are stored and used in various studies. Anonymisation is the most straightforward and common way for privacy protection of medical datasets by removing personal data for de-identification purposes. However, advanced re-identification techniques were developed and used to target the vulnerability of the anonymisation system by data mining companies, and data were then exploited by health insurance companies (Tanner, 2017). Thus, more rigorous data handling methods such as data decentralisation (storing data in separate locations) and federated machine learning (training algorithm across different decentralised local data) are necessary for institutes and companies dealing with large-scale personal data (Rieke et al., 2020). From patients and the general public’s perspective, there is an innate scepticism related to the use of AI for clinical applications, especially with limited understanding about how ML and personal data are used in medical research. Face-to-face communication between specialists and patients is effective in conveying the scope of ML applications and addressing questions and concerns in terms of patient satisfaction (Mirzaei and Kashian, 2020). Public education events such as interactive Patient and Public Involvement and Engagement (PPIE) activities can inform patients about how AI and ML research can lead to better disease management and how data are handled within a secured framework. With a better understanding of ML approaches and how personal data are stored, used and protected, patients are more likely to engage with such research.

The phenomenon of ML algorithm-driven discriminating decisions has been well-observed in other areas of research using AI, such as racial discrimination in criminal charge facial recognition technology (Perkowitz, 2021) and gender discrimination in job recruitment algorithms (Yarger et al., 2019). Algorithm discrimination is not exempt in the clinical world. For example, an implemented algorithm in the US healthcare system for future health care needs prediction is heavily biased against black patients because of the lack of data on these patients (Obermeyer et al., 2019). This algorithm-intrinsic bias is inherited from existing inequality in society as black patients are generally less accessible to the healthcare system. Another study showed that the predicted hospital mortality of patients in critical care can vary by up to 20% according to their ethnic group (Chen et al., 2018). Many inflammatory diseases are independently associated with demographic variables such as age, sex and ethnicity. For example, autoimmune diseases are more frequent in the female population (Gleicher and Barad, 2007), which sometimes, for practical reasons, promotes research only within the most represented groups of patients, discriminating against the under-represented ones. Moreover, model development is highly data-driven with low tolerance to missing values in model training, which can also lead to potential bias by not capturing the real-life patient population of interest. For example, previous studies showed that vulnerable populations are less likely to attend the same clinic regularly due to limited access to healthcare, including diagnostic testing and medicines (Arpey et al., 2017; Gianfrancesco et al., 2018). Unintentionally excluding these incomplete datasets will lead to development of models that are less effective in populations with existing disadvantages. Thus, it is important for researchers and data scientists representing the diversity of the human condition to have opportunities to participate in the decision making and algorithm supervision process, assessment of the underlying biases associated with AI and ML and implementation of regulatory adjustments. This will avoid the development of discriminating decision-aiding algorithms.

### The Future of Personalised Medicine

With such challenges evident at every possible step during the application of ML approaches, the ambition of personalised medicine to ensure that every individual receives an optimal treatment decision guided by their disease particularities and individual risk becomes uncertain. To warrant a future for ML applications in the clinical field, it is crucial to have universal procedure guidelines from data collection, data processing to model training, validation, and implementation (Figure 2). By ensuring the standardisation of ML applications, research study design can be optimised to facilitate granular and relevant data collection, as well as the use of an adequate sample size in relation to data multidimensionality to minimize the risk of significant data redundancy which can hamper the relevant patient identification (Plant and Barton, 2021). In addition, identification of reproducible biomarkers associated with response to therapy is one of the key requirements for personalized medicine approaches and we advocate for the use of truly independent data sets for validation. Although in theory, personalized medicine could be advanced by the use of ML algorithms for individual disease risk identification and prognostic, as well as therapy selection, its implementation in large health systems poses the ethical challenges of reconciling health risk inequalities with finite health care resources and standardised taxpayer or health insurance contributions (Rose, 2013). Future research should provide answers regarding the advantages of ML-driven personalised medicine strategies for long-term outcomes of patients in real-life.

## Conclusion

The versatility of ML applications allows researchers to tackle divergent unmet clinical needs of immune-mediated inflammatory disease with the most effective tools (Figure 1). Predictive ML models with outstanding biomarker selection capability are crucial for developing diagnostic and prognostic approaches with high sensitivity and accuracy, which are particularly useful in the early stages of the disease, as well as for the long-term disease management and selection of therapies at every disease stage. Patient stratification by unsupervised models and advanced drug development strategies supported by deep learning providing a more personalised treatment selection is especially relevant for patients with immune-mediated chronic inflammatory diseases, because of heterogeneity in clinical presentation, evolution and response to therapy. Despite several challenges which might impede some of the ML applications in clinical research and practice, the contribution of AI and ML techniques to personalised medicine for improved patient care is no doubt revolutionary.

## Glossary

Types of ML

Supervised Learning: The type of ML algorithms which generates predictive models based on labelled training data. Two main types of supervised learning include classification and regression, capable of predicting category and continuous output, respectively.
Unsupervised Learning: The type of ML algorithms which discovers underlying data structure based on unlabelled training data. Clustering is the main type of unsupervised learning.
Reinforcement Learning: The type of ML algorithms which sequentially self-correct from either positive or negative environmental feedback to maximise the model function.
Deep Learning: A subfield of machine learning that applies multiple layers of non-linear information processing for supervised or unsupervised feature extraction and transformation, using various neural network frameworks.

Main ML Model

Decision Tree: A tree-like predictive model going from observation to prediction result by repeatedly splitting the data into data subset based on selected variables. There are two main types of decision tree (classification tree, regression tree) which serves different purpose (predict category result or continuous result).
Random Forest: An ensemble classifier trained by a large number of unrelated decision trees. Bagging methods (or bootstrap aggregating) selects random samples from dataset when training each decision tree, which is applied in random forest models to improve model stability and accuracy.
Logistic Regression: A supervised classifier used to predict the probability of a binary variable.
Naive Bayes: A supervised classifier based on Bayes theorem. Naive Bayes models assume that the occurrence of a certain feature is independent of the occurrence of other features.
Support Vector Machine (SVM): A supervised learning model that builds a hyperplane in a high dimensional space for optimal separation between two classes, which can be used for classification and regression purposes.
K-Nearest Neighbours (kNN): A non-parametric classification algorithm which assigns the class of an unknown observation based on the class of a number (k) of similar observations in the feature space.
Artificial Neutral Network: Algorithms that mimic the neural networks of the human brain. The artificial neuron (node) in a neural network processed the received signals and transmit to connected neurons. A neural network contains layers of interconnected nodes, where signals travel from the first layer (input layer), through the hidden layers eventually to the last layer (output layer).

Performance Metrics

Classification Accuracy (CA): The rate of correct classifications (number of correct predictions divided by the total number of predictions).
Confusion Matrix: A 4x4 table showing the performance of a classification model. Rows represent the occurrences in the predicted class and columns represent the occurrences in the actual class.
Area Under Curve (AUC): An aggregated measure of performance of a binary classifier on all possible threshold values.
Precision: Performance metrics for specific class.

Fraction of correctly predicted occurrences in a specific predicted class

True Positive/(True Positive + False Positive).

Recall (Sensitivity): Fraction of correctly predicted occurrences in a specific actual class

True Positive/(True Positive + False Negative).

F1 Score: The harmonic mean of precision and recall.

2 × Precision × Recall/(Precision + Recall).

Gini Importance: The average gain of purity (model improvement) by splits of a given variable. Replacing a more important variables usually cause a larger decrease in Gini-gain. ML Model such as random forest uses “mean decrease in Gini importance” to measure the variable importance in generating the model performance.

Other Terms

Overfitting: Problem when a machine learning model fits too well to a particular dataset, causing it to lose generalization and predictive performance on other datasets.
Cross-Validation: Model validation method designed to estimate the model performance when predicting new data which is not in the original model training, to avoid problems such as overfitting or selection bias. K-fold cross-validation randomly partitioning the data into “k” complementary subset. “k-1” portions will be used in model training and the remaining portion for validation, and process repeats until all data is used in model training and validation.
